# Costs of accessing HIV testing services among rural Malawi communities

**DOI:** 10.1080/09540121.2018.1479032

**Published:** 2018-07-08

**Authors:** Linda Sande, Hendramoorthy Maheswaran, Collin Mangenah, Lawrence Mwenge, Pitchaya Indravudh, Phillip Mkandawire, Nurilign Ahmed, Marc d’Elbee, Cheryl Johnson, Karin Hatzold, Elizabeth L. Corbett, Melissa Neuman, Fern Terris-Prestholt

**Affiliations:** a Malawi-Liverpool-Wellcome Trust Clinical Research Programme, Blantyre, Malawi; b Faculty of Public Health & Policy, London School of Hygiene & Tropical Medicine, London, UK; c Institute of Psychology, Health and Society, University of Liverpool, Liverpool, UK; d The Centre for Sexual Health and HIV AIDS Research (CeSHHAR), Harare, Zimbabwe; e Zambart, Lusaka, Zambia; g Population Services International, Lilongwe, Malawi; h Department of HIV/AIDS, World Health Organisation, Geneva, Switzerland; i Population Services International, Harare, Zimbabwe; j Faculty of Infectious and Tropical Diseases, London School of Hygiene & Tropical Medicine, London, UK; k Faculty of Epidemiology and Population Health, London School of Hygiene & Tropical Medicine, London, UK

**Keywords:** HIV, HIV testing and counseling, total costs

## Abstract

HIV testing is free in Malawi, but users may still incur costs that can deter or delay them accessing these services. We sought to identify and quantify these costs among HIV testing service clients in Malawi. We asked residents of communities participating in a cluster randomised trial investigating the impact of HIV self-testing about their past HIV testing experiences and the direct non-medical and indirect costs incurred to access HIV testing. We recruited 749 participants whose most recent HIV test was within the past 12 months. The mean total cost to access testing was US$2.45 (95%CI: US$2.11–US$2.70). Men incurred higher costs (US$3.81; 95%CI: US$2.91–US$4.50) than women (US$1.83; 95%CI: US$1.61–US$2.00). Results from a two-part multivariable regression analysis suggest that age, testing location, time taken to test, visiting a facility specifically for an HIV test and district of residence significantly affected the odds of incurring costs to testing. In addition, gender, wealth, age, education and district of residence were associated with significant user costs.

## Introduction

Eastern and Southern Africa account for the highest numbers of people living with HIV (PLHIV), newly infected with HIV, and dying from HIV (UNAIDS, 2017). HIV testing is an essential gateway to HIV prevention, treatment, care and support services since receipt of an HIV diagnosis empowers individuals to make informed decisions about follow on services in the cascade (World Health Organization, 2015; World Health Organization & UNAIDS, 2017). The global entities involved in AIDS eradication have adopted ambitious treatment targets: by 2020, 90% of all PLHIV will know their HIV status, 90% of all people with diagnosed HIV infection will receive sustained antiretroviral therapy (ART) and 90% of all people receiving ART will have viral suppression (UNAIDS, 2014a). Ensuring that 90% of PLHIV are aware of their status will support enrolment in HIV care and achievement of these global treatment goals (UNAIDS, 2014a).

However, despite impressive efforts in scaling-up availability of HIV testing and treatment services in the region, including freely available HIV testing at nearly all healthcare settings, testing uptake remains inadequate to reach the global goals (Church et al., 2017). Malawi has been leading the way in scaling-up HIV services (Lowrance et al., 2008; UNAIDS, 2014b) but an estimated 35% of men and 18% of women have never tested for HIV and 60% of young people aged 15–19 years have never tested (CDC & GoM, 2017). Uptake of HIV testing also remains low amongst poorer individuals and those with less formal education (Kim, Skordis-Worrall, Haghparast-Bidgoli, & Pulkki-Brännström, 2016).

Previous studies in sub-Saharan Africa have cited location, distance, waiting time, costs, confidentiality concerns, low perceived risk and infrequent contact with the health-care system as barriers to accessing HIV testing (Angotti et al., 2009; Morin et al., 2006; Musheke et al., 2013; Sharma, Ying, Tarr, & Barnabas, 2015). Individuals often incur substantial access costs when utilising public sector HIV testing and treatment services even when they are provided free at point of use (Chimbindi et al., 2015; Lubega et al., 2013; Maheswaran et al., 2016; Pinto, Lettow, Rachlis, Chan, & Sodhi, 2013).

In urban settings, HIV testers incur costs close to twice their daily earning incomes (Maheswaran et al., 2016). These costs are likely to be higher in more rural settings, however little is known about these costs and whether these vary by different population groups or testing modalities, which limits efforts to minimise or offset testing costs to increase uptake. Awareness of costs incurred by rural HIV testers is particularly important since 84% of the Malawi population is rural with 57% of the rural population classified as poor compared to 17% of the urban population (International Monetary Fund, 2017; World Bank, 2014). The poor in developing countries like Malawi are even less likely than the better off to receive effective health care with existing costs barriers proposed as one of the deterrents of this low use (O'Donnell, 2007; Russel, 2004).

The World Health Organisation (WHO) guidelines have highlighted the need for strategic approaches to deliver HIV testing services (HTS) (World Health Organisation, 2016). HIV self-testing (HIVST) and community-based HIV testing are proposed as having the potential of increasing testing uptake especially for men, key populations and young people who would not normally access HIV testing services (Malawi Ministry of Health, 2016; World Health Organisation, 2016). Young people for instance, have previously demonstrated an aversion to price due to their limited access to resources (Indravudh et al., 2017; Sibanda, Maringwa, et al., 2017). Research on these costs is essential to appropriately targeting these sub-populations lagging behind in access to testing.

In this study, we sought to examine (1) the costs borne by users of HIV testing services in rural Malawi; (2) whether certain population subgroups incur higher costs; and (3) whether costs differ based on the mode of testing. To the best of our knowledge, this is the first study to identify and quantify specific costs of HIV testing in a rural setting. Other studies in the region have explored determinants of testing (Camlin et al., 2016; Helleringer, Kohler, Frimpong, & Mkandawire, 2009; Lépine, Terris-Prestholt, & Vickerman, 2014), costs of providing HIV services (Maheswaran et al., 2016; Mangenah, Mwenge, et al., 2017; Mwenge et al., 2017; Sharma et al., 2015), and costs of accessing tuberculosis (TB) treatment (Kemp, Mann, Simwaka, Salaniponi, & Squire, 2007) and ART (Bergmann, Wanyenze, & Stockman, 2017; Chimbindi et al., 2015; Pinto et al., 2013; Rosen, Ketlhapile, Sanne, & DeSilva, 2007). The few that have explored costs associated with HIV testing have either focused on urban settings (Maheswaran et al., 2016) or examined costs without considering lost income (Bergmann et al., 2017). The results of this study will inform the design of future HIV testing services and interventions aimed at overcoming financial barriers to testing.

## Methods

### Study setting and design

HIV testing in Malawi is freely provided. Individuals may voluntarily access HIV testing at a health facility; may be advised to test by a health professional [provider-initiated testing and counseling (PITC)]; may be offered testing as part of routine antenatal care (ANC) (accessed by both the pregnant women and their accompanying male partners) or TB care (also a form of PITC); or may have access to community-based HIV testing services (CBHTS) including through testing campaigns and outreach, home-based or door-to-door testing, workplace testing, mobile testing, and testing through educational institutions.

**Table 1.** Descriptive statistics.

Variable	Regression Inclusion	Expected Direction
Gender	Indicator:	
	­ Men (reference group) ­ Women	Men are expected to incur higher costs than women to reflect their higher earning potential relative to women
Age (Years)	Indicator:	
	­ 16–19 Years; 20–24 Years; 25–39 Years; 40–64 Years; 65+ Years	Financial productivity is expected to increase with age starting from age 20 hence raising the opportunity cost to testing up to age 65
Education	Indicator:	
	­ No Formal education (reference group) ­ Incomplete Primary education ­ Some Secondary Education ­ Complete Secondary Education or higher	Education as a proxy for earning potential, implying that the higher the level of education the higher the cost for testing
Number of Children	Continuous: The participant’s number of children	Number of children is positively associated with any child care costs a participant might have incurred while accessing testing hence increasing the total costs incurred
Test Location	Indicator:	
	­ Facility-Based Testing (reference group) ­ Community HTC ­ Other Place	Community-based HTC reduces logistic barriers hence lowers the opportunity cost of testing. Other place testing depends on where the person tested for example, if at home testing e.g., self-testing then lower costs than facility-based testing
Amount of Time Taken to Receive Testing	Continuous: Time taken (including travel) in hours to access HIV testing	The more time taken away from work to seek testing, the higher the cost of testing through lost income
Reason for visiting Testing Centre	Indicator:	
	­ Had other reasons for visiting a testing centre aside from HIV testing (reference group) ­ Visited a testing centre specifically for an HIV test	Visiting a testing centre for other reasons aside from HIV testing has potential of economies of scope hence reduced total costs
Wealth Index	Indicator:	
	­ Households are ranked into wealth quintiles with the poorest as the reference group	Wealth is a proxy for ability to pay; the higher the wealth quintile, the higher the participant’s expenditure to access testing
District of Residence	Indicator:	
	­ Blantyre District (Reference Group) ­ Machinga District ­ Mwanza District ­ Neno District	There should not be difference in costs of testing by district

We undertook a baseline household survey as part of a cluster-randomised trial (CRT) investigating the impact of community-based distribution of HIVST in rural Malawi (ClinicalTrials.gov Identifier: NCT02718274). The CRT was conducted in rural villages of Blantyre, Machinga, Mwanza and Neno in Southern Malawi. The CRT comprised a population of approximately 62,500 residents with 22 clusters defined by the service catchment area of public primary health facilities with active ART clinics. The HIV prevalence in the four districts was approximately 11% (National Statistics Office & ICF Macro, 2017).

Within each cluster, villages were selected for inclusion in the baseline survey based on location, population size, road accessibility and presence of pre-existing reproductive health community-based distribution agents. Households in these evaluation villages were randomly sampled for a baseline household survey which was conducted between May and August 2016. The sampling of the survey ensured inclusion of at least 250 adults per cluster, with the sample size calculated based on the primary outcome of the trial. All household members aged 16 years or older were eligible to participate in the survey. Details on the sample size calculation for the main trial can be found in the trial protocol available at http://hivstar.lshtm.ac.uk/.

Research assistants visited selected households and administered an electronic, face-to-face, questionnaire to all household members aged above 16 years who agreed to participate. The main questionnaire included questions about sociodemographics and HIV testing history. Due to time and resource constraints, an extended questionnaire was administered to a random 20% subset of participants responding to the main questionnaire. The extended questionnaire included questions on the costs of HIV testing as well as other questions on health care utilisation and stigma.

### Assessing costs and location of HIV testing

Participants who reported testing within the previous 12 months were asked the location of testing, including whether facility- or community-based; if their most recent test was accessed separately from other health services or as part of antenatal care ANC or PITC; total time taken to access HIV testing; and the direct non-medical and indirect costs they incurred. The 12 months recall period is in line with other studies on health care use and/or out-of-pocket expenditure (van Doorslaer & Masseria, 2004; Heijink, Xu, Saksana, & Evans, 2011) and a similar recall period is used to collect household non-food expenditures in the Malawi integrated household survey which is a major socio-economic survey conducted by the Malawi National Statistical Office. It is worth noting that there is no general answer to the question of optimal recall period with the choice dependent on the primary objective of the data collection (Clarke, Fiebig, & Gerdtham, 2008).

We derived a list of potential costs based on the literature and previous work undertaken in Malawi to inform development of the study questionnaire (Kemp et al., 2007; Maheswaran et al., 2016; Pinto et al., 2013). We asked participants how much they had paid for the round trip to the testing facility (transport cost), and if they had paid any consultation or service fees (consultation cost) related to testing (sometimes incurred at private facilities), excluding any fees for other services they accessed at the same time. Participants were also asked if they spent money on any food and drink items (food costs) while accessing testing and, if so, how much they spent. Additionally, we asked participants about any costs they might have incurred by paying a caretaker to watch their children for the time they sought testing (child care costs), and about any other costs they might have incurred as they sought testing (other costs). We further asked participants to approximate the amount of money they would have earned during the entire time they took to access testing (lost income).

### Other covariates

Participants were also asked questions on socio-demographics (age, gender and education), the number of children they have and ownership of eight household assets.^1^ We estimated household wealth using the principal component analysis (PCA) method, with household assets as a proxy for wealth (Filmer & Pritchett, 2001), and we further classified wealth into quintiles. Table 1 further summarises all the covariates.

Ethical approvals were obtained from the College of Medicine Research Ethics Committee in Malawi and the Research Ethics Committee of the London School of Hygiene and Tropical Medicine. We obtained written informed consent from all participants in the extended questionnaire before their interview.

### Statistical methods

All analysis was undertaken in STATA version 14.0 (Stata Corporation, Texas, USA). Costs were estimated in 2016 Malawi Kwacha (MWK) and converted to 2016 US dollars at an exchange rate of MWK 729.89/US$ (Reserve Bank of Malawi, 2017).

Cost data were categorised into direct non-medical costs and indirect costs. Direct non-medical costs included those directly incurred by participants and indirect costs refer to productivity and income losses due to accessing testing services. We include data for the entire sample who had complete cost data and present it using means with 95% confidence intervals. To assess the burden imposed on participants, we compared their total direct non-medical and indirect costs with the national poverty line of US$1.20/day. The poverty line was adopted from the Third Malawi Integrated Household Survey (IHS) of 2011, converted to US$ at the average 2011 exchange rate of MWK162.84/US$ (National Statistics Office, 2012; World Bank, 2018) and adjusted for inflation using the national gross domestic product (GDP) deflator for 2011 of 14% (World Bank, 2018).

To determine the significant predictors of costs, we estimated a multivariable two-part model (TPM). Individual-level user cost data pose estimation challenges since individual-level medical expenditures or costs of treatment typically feature a spike at zero and are strongly skewed with a heavy right-hand tail (Jones, 2010). There is no unique way to deal with these estimation challenges associated with cost data with literature recommending that the choice of appropriate estimation approach should be determined by the research questions and the characteristics of the data (Buntin & Zaslavsky, 2004; Diehr, Yanez, Ash, Hornbrook, & Lin, 1999; Gregori et al., 2011; Griswold, Parmigiani, Potosky, & Lipscomb, 2004). The common proposed estimation approaches are the log-transformed OLS, Tobit model, TPM and generalised linear models (GLM) with a log-link function (Buntin & Zaslavsky, 2004; Gregori et al., 2011; Griswold et al., 2004; Jones, 2010; Nichols, 2010).

A Tobit regression model and a TPM were better fit for our data as they are both able to handle excess zeroes and positive distribution associated with cost data (Jones, 2010). GLM and log-transformed ordinary least squares (OLS) on the other hand, do not take into account the excess zeroes in the data and therefore generates biased estimates. We therefore, estimated a log-transformed Tobit and a TPM with a logit model for the first part and log-transformed OLS regression for the second part. Given our main objective, a TPM is the appropriate estimation approach as it can distinguish the probability of incurring costs for testing and assess significant cost drivers for those who incurred costs.

To account for the clustering of the data by district, a fixed effect approach was used. We then applied a likelihood ratio test to identify the most parsimonious model between the restricted and unrestricted TPM models. We further identified the most appropriate functional form for age (testing for non-linearity) using the likelihood-ratio test and did not find significant justification for this quadratic relationship.

We explored socio-demographic and socio-economic variables and accessibility of testing centres as determinants of total costs:

$$\begin{array}{r} \ln(\text{Total}\;\text{Cost}{\text{s}}_{i}+1)=f\left[\begin{array}{c} District,\;\;\text{Gender, }\;\text{Wealt}{\text{h}}_{\mathrm{hh}},\;Age\;\text{categories, }\;\text{Education, }\;\text{Number}\;\text{of}\;\text{Children, }\;\\ \text{Time}\;\text{Taken ( Hours) , }\;\text{Reason}\;\text{for}\;\text{visiting}\;\text{testing}\;\text{centre}\; \end{array}\right] \end{array}$$

To reduce the skewness in the cost data, we modelled the costs using a log transformation. We log transformed user costs as $\ln(\text{Total}\;\text{Cost}{\text{s}}_{i}+1)$ as suggested by the literature (McCune, Grace, & Urban, 2002). Table 1 summarises the a priori direction of association of the determinants.

## Results

### Participants’ characteristics

A total of 5551 participants were recruited into the baseline survey and 1388 responded to the extended questionnaire. Seven hundred and forty-nine (14%) participants reported having had at least one HIV test in the previous 12 months, making them eligible for this sub-study. Baseline characteristics of these 749 participants are presented in Table 2. In brief, 32% of the participants were men, 33% of the participants were aged 16–24 years and 18% had no formal education. Most of the participants (83%) reported facility-based testing as their most recent testing approach. Among those who tested in a facility, more participants (76%) accessed testing through PITC. In addition, men reported spending an average of 2.9 h and women reported spending an average of 3.5 h to access testing services.

**Table 2.** Participant characteristics (*n* = 749)^a^.

		Men (*n* = 237, 32%)		Women (*n* = 512, 68%)	
	*N*	Percentage	*N*	Percentage	
Age (Years)	16–19	23	9.8%	52	10.2%
	20–24	35	14.8%	135	26.4%
	25–39	96	40.7%	205	40%
	40–64	63	26.7%	102	19.9%
	65+	19	8.1%	18	3.5%
Education	No formal Edu.	19	8.0%	112	21.9%
	Primary Edu.	160	67.5%	331	64.7%
	Some Secondary Edu.	38	16.0%	57	11.1%
	Complete Secondary or Higher Edu.	20	8.4%	12	2.3%
Wealth Index^b,c^	Lowest Quintile	64	27.0%	227	44.3%
	2nd Lowest Quintile	40	16.9%	57	11.1%
	Middle Quintile	28	11.8%	69	13.5%
	2nd Highest Quintile	45	19.0%	70	13.7%
	Highest Quintile	60	25.3%	89	17.4%
Test Location	Hospital/Clinic/Health Centre	148	62.5%	295	57.6%
	ANC Clinic	17	7.2%	106	20.7%
	VCT Centre	24	10.1%	31	6.1%
	Community/Mobile HTC	47	19.8%	74	14.5%
	Other Testing Place	1	0.42%	6	1.1%
Number of Children	Mean (min-max)	3	(0–12)	3	(0–13)
Reason for facility visit	HIV Test	168	70.9%	283	55.3%
	HIV Test + Other Services	69	29.1%	229	44.7%
Time Taken	≤1 h	73	30.8%	104	20.3%
	1–3 h	83	35.0%	181	35.4%
	3–6 h	66	27.9%	182	35.6%
	>6 h	15	6.3%	45	8.8%
District	Blantyre	62	26.2%	147	28.7%
	Machinga	70	29.5%	172	33.6%
	Mwanza	30	12.7%	51	10%
	Neno	75	31.7%	142	27.7%

^a^3 Participants had incomplete data.

^b^Wealth index estimated through undertaking principal component analysis of responses to asset ownership and housing environment.

^c^Assets selected in the baseline data did not do well in differentiating the poorest from one another.

### Direct non-medical and indirect costs

Direct non-medical and indirect costs stratified by gender and cost-category are summarised in Table 3. Twenty percent of the participants incurred zero costs for testing. The median cost for participants who incurred costs was US$2.06. The mean total cost per participant was US$2.45 (95%CI: US$2.11–US$2.70) with lost income accounting for 83% of the total costs. Men incurred higher mean total costs than women: US$3.81 (95%CI: US$2.91–US$4.50) versus US$1.83 (95%CI: US$1.61–US$2.00).

**Table 3.** Direct non-medical and indirect costs by gender and cost category.

		Men (US$)		Women (US$)	Total Sample (US$)		
Cost Category		Mean (95% CI)	% of Men	Mean (95% CI)	% of Women	Mean 95% CI	% of Total Sample
Direct non-medical costs	Transport	0.25 (0.15–0.36)	6.6%	0.16 (0.11–0.22)	8.7%	0.19 (0.14–0.24)	7.8%
	Consultation	0.03 (0.00–0.05)	0.8%	0.03 (0.01–0.04)	1.6%	0.03 (0.01–0.04)	1.2%
	Food	0.18 (0.14–0.22)	4.7%	0.13 (0.10–0.15)	7.1%	0.14 (0.12–0.17)	5.7%
	Other	0.05 (0.02–0.09)	1.3%	0.02 (0.01–0.04)	1.1%	0.03 (0.02–0.05)	1.2%
Indirect Costs	Child Care	0.06 (0.02–0.11)	1.6%	0.01 (0.00–0.03)	0.6%	0.03 (0.01–0.05)	1.2%
	Lost Income^a^	3.24 (2.45–4.03)	85.0%	1.48 (1.31–1.65)	80.9%	2.03 (1.75–2.31)	82.9%
Total direct non-medical and indirect cost		3.81 (2.91–4.50)	100%	1.83 (1.61–2.00)	100%	2.45 (2.11–2.70)	100%

^a^Lost Income had a median cost of US$1.37; US$2.06 for men and US$0.96 for women.

### Cost determinants

The logit component of the TPM demonstrated that age, testing location, time taken to acquire a test, visiting a facility specifically for an HIV test and district of residence significantly affected the odds of incurring costs for testing. The odds of incurring testing costs are 18% higher for participants aged between 25–39 years than participants aged between 16–19 years. In addition, participants who tested within their communities (mobile testing) had 61% lower odds of incurring costs than participants who tested at facilities. Each additional hour spent seeking testing increased the odds of incurring costs by 48%. Participants who visited a testing site specifically for an HIV test had 48% higher odds of incurring costs for testing than those who accessed testing in addition to other health care services. And finally, residence in Mwanza district was associated with 95% higher odds of incurring costs when compared to residence in Blantyre district (Tables 4 and 5).

**Table 4.** Multivariable analysis of log-transformed Tobit regression model (Dependent Variable: total direct non-medical and indirect costs).

	Determinants (Reference Category)	Coefficient	95% CI	*P*-value
Gender	(Male)			
	Female	−0.323***	(−)0.457–(−)0.189	0.000
Wealth	(Lowest Quintile)			
	2nd Lowest Quintile	−0.049	(−)0.239–0.141	0.613
	Middle Quintile	0.169*	(−)0.024–0.362	0.086
	2nd Highest Quintile	0.003	(−)0.176–0.182	0.975
	Highest Quintile	0.175**	0.007–0.343	0.041
Age (Years)	(16–19)			
	20–24	0.411***	0.178–0.643	0.001
	25–39	0.640***	0.406–0.873	0.000
	40–64	0.685***	0.395–0.974	0.000
	65+	0.195	(−)0.169–0.56	0.293
Education	No Formal Edu.			
	Primary Edu.	0.013	(−)0.151–0.177	0.877
	Incomplete Secondary Edu.	0.253**	0.017–0.489	0.036
	Complete Secondary or Higher	0.530***	0.198–0.863	0.002
Children	No. of Children	0.000	(−)0.033–0.034	0.982
Testing Location	Facility			
	Community	−0.396***	(−)0.571–(−)0.220	0.000
	Other	−0.175	(−)0.858–0.508	0.614
Time Taken	Time (Hours)	0.049***	0.023–0.077	0.000
Reason for visiting	HIV Test + Other			
	HIV Test	0.079	(−)0.045–0.204	0.211
District	Blantyre			
	Machinga	0.059	(−)0.097–0.214	0.460
	Mwanza	0.350***	0.139–0.560	0.001
	Neno	−0.007	−0.164–0.149	0.927
	Constant	0.208	(−)0.113–0.529	0.164
	Observations			746^a^

Note: ***p < 0.01, **p < 0.05, *p < 0.1.

^a^3 observations had incomplete data.

**Table 5.** Multivariable analysis of Two-Part Model on total direct non-medical and indirect costs with first part (logit) and second part (Log-transformed OLS).

Determinants (Reference Category)		Two-Part Model	
	logit	Log-transformed OLS	
Gender	(Male)		
	Female	−0.221	−0.517***
Wealth	(Lowest Quintile)		
	2nd Lowest Quintile	−0.196	−0.0113
	Middle Quintile	−0.108	0.398***
	2nd Highest Quintile	−0.168	0.0644
	Highest Quintile	0.342	0.161
Age (Years)	(16–19)		
	20–24	0.468	0.610***
	25–39	0.777**	0.964***
	40–64	0.674	1.031***
	65+	−0.323	0.736***
Education	(No Formal Edu.)		
	Primary Edu.	0.177	−0.0569
	Incomplete Secondary Edu.	0.430	0.248
	Complete Secondary Edu.	0.951	0.628***
Number of Children	No. of Children	0.0604	−0.0164
Testing Location	(Facility)		
	Community testing	−0.946***	−0.204
	Other	−0.820	0.0617
Time Taken	Time (Hours)	0.203***	0.0161
		(0.0530)	(0.0197)
Reason for visiting	(HIV Test + Other)		
	HIV Test	0.393*	0.0374
District	(Blantyre)		
	Machinga	0.253	0.0857
	Mwanza	0.666*	0.434***
	Neno	−0.190	0.0594
	Constant	−0.0902	−0.118
	Observations	746^a^	746^a^
Pseudo *R*^2^		0.116	
Adjusted *R*^2^			0.1579
Log Likelihood		−335.04519	−847.03399

Note: ***p < 0.01, **p < 0.05, *p < 0.1.

^a^3 observations had incomplete data.

On the other hand, the log-transformed OLS component of the TPM demonstrated that gender, age, wealth, education and district of residence was associated with significant user costs. Holding everything else constant, men on average incurred 52% higher costs for testing than women.

Older age groups incurred significantly higher costs than the 16–19 age group. Participants aged between 20–24 years; 25–39 years incurred 61% and 96% higher costs respectively, than participants aged between 16–19 years. Participants aged between 40–64 years and 65+ years on average incurred more than double and 74% higher costs respectively, than participants aged between 16–19 years. There was no difference in average testing costs among participants with lower than complete secondary education and those without any formal education. However, participants with complete secondary education or higher on average incurred 63% higher costs than those with no formal education. Finally, participants in Mwanza district incurred on average 43% higher costs than participants resident in Blantyre district.

## Discussion

This study examined the costs borne by users when accessing HIV testing services in rural villages of Southern Malawi. Our findings indicate that the average cost of accessing HIV testing in rural Malawi is less than that reported in urban areas of the country (US$3.09 per test) (Maheswaran et al., 2016), yet rural testers incur costs that are equivalent to twice the daily minimum income required for their basic needs (national poverty line at US$1.20 a day) (National Statistics Office, 2012). In a country where at least 51% of the population live below the national poverty line and 71% live below the international poverty line of US$1.90 a day (National Statistics Office, 2012; World Bank, 2014), these costs are likely to be prohibitive for a large proportion of the population.

Our study also demonstrated that there are significant average cost differences between men (US$3.81) and women (US$1.83). Historically, there has been low uptake of HIV testing and poor linkage into care amongst men relative to women, particularly in sub-Saharan Africa (Camlin et al., 2016). It is likely that these high costs have contributed to the lower uptake. Seeking testing imposes both a direct non-medical cost but also the lost opportunity cost of hours away from productive activities (Angotti et al., 2009; Ganesh, 2015; Musheke et al., 2013; Wolff et al., 2005). Our findings show that these opportunity costs comprise a significant proportion (83%) of the total testing costs in this population. For most, the prospect of learning their HIV status may not be a sufficient incentive to bear these costs (Angotti et al., 2009), unless they are already sick. This is further evidenced by the large proportion of men in our sample who accessed testing through PITC (70%) and very few who voluntarily attended facilities for the sole purpose of learning their HIV status (10%), suggesting that most men in rural Malawi access testing as an add-on to other health care services, rather than seeking out testing independently.

The large proportion of total costs associated with lost income was driven by long travel times and long waiting times at testing facilities. On average, participants spent three hours to access HIV testing services, with men spending less time (2.9 h) than women (3.5 h). Similar long wait times (3.4 h) were observed among adults utilising public sector HIV and TB services in South Africa (Chimbindi et al., 2015). Taking measures to improve efficiency at HIV testing facilities, such as increasing staffing for this service, could reduce waiting times and therefore reduce the time taken from employment and other activities.

Delivering HIV testing closer to people’s homes or at times convenient to users may also mitigate financial barriers to testing. We found that community-based testing is associated with a lower probability of incurring costs than facility-based testing, therefore decentralising testing services beyond static facilities may be necessary to increase uptake. The popularity, especially among men, of community-based HIV testing and HIVST models has been previously demonstrated (Angotti et al., 2009; Choko et al., 2015; Morin et al., 2006; Mwenge et al., 2017; Sebapathy, Van den Bergh, Fidler, Hayes, & Ford, 2012; Sharma et al., 2015; World Health Organization, 2015). HIVST and other home-based testing can be advantageous in that they substantially reduce or completely eliminate costs borne by users when testing (Maheswaran et al., 2016; Sharma et al., 2015).

Financial and non-financial incentives also offer an alternative to reducing or offsetting testing costs and promoting uptake. Small non-monetary incentives are associated with significantly increased community testing and HIV case diagnosis (Sibanda, Tumushime, et al., 2017). It is worth noting that although small financial incentives have been effective in increasing health care uptake (Choko et al., 2017; Mangenah, Sibanda, et al., 2017; Pettifor, MacPhail, Nguyen, & Rosenberg, 2012), different amounts of incentives have different levels of effectiveness. Incentives that cover transport and opportunity costs are generally associated with better testing and linkage to care than incentives equivalent to transport reimbursement only (Choko et al., 2017).

## Study limitations and strengths

Our study used retrospective interviews to collect expenditure data for participants’ most recent HIV test. This approach introduces potential for recall bias. We limited this recall bias by recruiting participants with an HIV test within a period of 12 months preceding the interview. In addition, there is potential for downward bias of the testing costs because individuals with prohibitively high expected costs will not have tested. Our follow-up research will explore more advanced statistical models to reduce this downward bias.

Despite these limitations, our study adds valuable information to the literature on access to HIV testing. Unlike previous studies, we included lost income as a cost to testing which enabled us to determine the full economic burden of testing on users in a rural setting.

## Conclusion

Though HIV testing services are “free” in Malawi, users incur costs to access these services in rural parts of the country that are double the national poverty line. In these contexts, men incur higher costs to access HIV testing services than women, with lost income as the largest cost component. Increasing uptake of testing services, especially for men, will likely require bringing testing services closer to the communities, improving efficiency of facility-based testing and potentially introducing financial or non-financial incentives as a way to motivate uptake and offset the total costs associated with this portion of the HIV cascade.

## Note

1 Asset index: Electricity, radio, working television set, mobile phone, landline telephone, refrigerator and bed with mattress.
